# CyberKnife Radiosurgery of Skull-base Tumors: A UK Center Experience

**DOI:** 10.7759/cureus.2380

**Published:** 2018-03-27

**Authors:** Hannah P Wilson, Patricia M Price, Keyoumars Ashkan, Andrew Edwards, Melanie M Green, Timothy Cross, Ronald P Beaney, Rhiannon Davies, Amen Sibtain, Nick P Plowman, Christy Goldsmith

**Affiliations:** 1 Department of Surgery and Cancer, Imperial College London, London, GBR; 2 Department of Radiation Oncology, The Harley Street Clinic, London, GBR; 3 Department of Neuro Oncology, Guy’s and St. Thomas’ Hospital, London, GBR; 4 Clinical Oncology, Guy's and St. Thomas' Hospital, London, GBR

**Keywords:** cyberknife, intracranial, skull base, tumors, treatment outcomes, stereotactic, radiosurgery, fractionated radiotherapy

## Abstract

The study aim was to evaluate patient individualized Cyberknife® treatment for heterogeneous skull-base tumors. Patients treated between 2009 and 2013 at The Harley Street Clinic were studied. In total, 66 patients received 15–30 Gy in 1–5 fractions to a median planning target volume (PTV) of 6.4 cc, including patients with secondary, multiple, residual and recurrent tumors, and those with tumors of uncertain pathological type. Outcome analysis was pragmatically restricted to 35 patients who had single, primary tumors treated with curative intent, and sufficient diagnostic and outcome information. Sixteen vestibular schwannoma patients with median PTV 3.8 cc (range 0.81–19.6) received 18–25 Gy in 3–5 fractions: 81% showed no acute toxicity, 50% reported no late toxicity, 71% of symptoms were stable/improved and local control was 100% at 11.4 months median follow-up. Twelve meningioma patients with median PTV of 5.5 cc (range 0.68–22.3) received 17–30 Gy in 1–5 fractions: 83% experienced no acute toxicity, 33% reported no late toxicity, 88% of symptoms were stable/improved and local control was 100% at 22.1 months median follow-up. Seven patients with other tumor types with median PTV of 24.3 cc (range 7.6–100.5) received 15–28.5 Gy in 1–5 fractions: 57% experienced no acute toxicity, 57% reported no late toxicities, 66% of symptoms were stable and local control was 43% at 14.9 months median follow-up. When tumor types were considered together, smaller tumors (PTV < 6.4 cc) showed reduced acute toxicity (p = 0.01). Overall, smaller benign tumors showed low acute toxicity, excellent local control, and good symptom management: a focus on enhanced neurological preservation may refine outcomes. For other tumor types outcome was encouraging: a focus on optimal dose and fractionation scheduling may reduce toxicity and improve local control. Individual patient experiences are detailed where valuable lessons were gained for optimizing local control and minimizing toxicity.

## Introduction

Intracranial tumors of the skull-base, including cerebellopontine angle (CPA) tumors, are typically difficult to access surgically and are frequently located within or proximate to critical structures such as cranial nerves or brainstem, such that surgical removal may be impossible, incomplete, or carry high risk of neurological injury. The introduction of radiosurgery has therefore altered the landscape of treatment practice for intracranial lesions. Multiple retrospective studies undertaken outside the UK have shown safety and efficacy of intracranial radiosurgery and demonstrated its utility as a primary or adjuvant treatment approach [1-18]. The recent availability of advanced radiosurgical techniques has therefore improved treatment options for patients with skull-base tumors in the UK.

CyberKnife® (Accuray, Sunnyvale, USA) is a frameless robotic radiosurgical treatment platform [19]. The associated 6D Skull Tracking system enables highly conformal intracranial high-dose tumor targeting, with sub-millimetre precision beam delivery and rapid dose fall-off achievable around the contoured tumor. An additional benefit is the ability to easily fractionate treatment to minimize toxicity—as per radiobiological principles—particularly when the radiation tolerance of adjacent organs-at-risk (OAR) is paramount [20]. To assure safe and optimized radiation delivery, multi-disciplinary team management is necessary for optimal patient selection and patient individualized treatment planning.

The primary goals of skull-base radiosurgery are maximal tumor control and optimal functional outcome with minimal morbidity risk. Although there are currently some practice guidelines, optimal treatments are still evolving. There are currently considerable variations within and between treatment centers from patient selection through to treatment delivery, yet there is limited availability of detailed outcome data for treatment comparison to shape best practice. Moreover, although overall safety and efficacy has been demonstrated, this has largely been in smaller, benign tumors where good local control and acceptable toxicity has been achieved. Reduction of treatment morbidity and improved symptom management have therefore become increasingly important in the ongoing process of treatment refinement for these tumors [21,22]. For larger, malignant tumors, the outcome is generally less predictable: although both tumor type and size have been reported to influence the outcome for base-of-skull radiosurgery [1-7, 10-14, 18,23]. This reflects inherent variable characteristics such as tumor histo-biological radiosensitivity, anatomical location, the geometric proximity of adjacent OAR, and limitations of the radiosurgical platform. To support efforts in treatment refinement, it is therefore important to examine patient and tumor factors, and variations to patient selection, prescription and planning methods, that influence efficacy and toxicity, the preservation of neurological function, and post-treatment symptom resolution.

Here we report the first UK experience of CyberKnife® radiosurgery applied as primary, adjuvant, recurrence or palliative treatment for patients with all types of skull-base tumors. For the purposes of pragmatic comparative outcome analysis in such a broad heterogeneous patient group, retrospective analysis of patient toxicity, symptomatic status, local control, and survival was confined to patients with single primary tumors of known histopathological type who were treated with curative intent.

## Materials and methods

Study approval and consent

This work was carried out within the auspices of HCA International—with the approval of HCA Healthcare UK executives as part of ongoing treatment review and research and development processes—to assure the highest quality standards for patient treatment. All patients gave informed written consent for treatment and it is mandated policy of HCA Healthcare UK to audit clinical outcomes within their quality, safety, and clinical governance frameworks. For the purposes of subsequent analysis of patient treatment data, patient information was anonymized to avoid bias and ensure that the study abided by the principles of the Declaration of Helsinki.

Patients

Consecutive patients with tumors located at the skull-base who received radiosurgery between 2009 and 2013 at The London CyberKnife® Centre, The Harley Street Clinic, were studied. Patient suitability for radiosurgery was determined by multi-disciplinary team discussion of clinical and radiological findings, incorporating the expert opinion of radiation oncologists, neuroradiologists, and neurosurgeons. Patient, tumor, and treatment information were recorded at the time of treatment and post-treatment outcome and toxicity measures were subsequently clinically assessed. For the purposes of pragmatic comparative outcome analysis in such a broad heterogeneous patient group, the outcome analysis cohort was defined retrospectively to include patients with single primary tumors of the skull-base, who were treated with curative intent, and for whom reliable diagnostic tumor information was available. Patients with multiple tumors, secondary metastatic tumors, pathologically uncertain tumors, or those who received treatment with palliative intent, were excluded from outcome analysis.

Treatment technique

Patients underwent computed tomography (CT) simulation using a GE LightSpeed 16 slice scanner (GE Healthcare, Buckinghamshire, UK). CT scans were performed helically with axial slice thickness of 1.25 mm. Patients were positioned supine on a memory foam mattress with a headrest, knee supports and arms by their sides. Immobilisation was achieved with a 2.4 mm thermoplastic mesh covering the skull and face (Civco, Sunnyvale, USA). Planning magnetic resonance imaging (MRI) scans were acquired with an axial thickness of ≤2 mm following administration of gadolinium contrast (where appropriate) to aid target definition. Planning MRI images were fused with planning CT scans to optimally define the tumor target and normal tissues.

Image fusion, target delineation and treatment planning were performed using MultiPlan® software system (Accuray Inc., Sunnyvale, USA). Clinical target volumes (CTV) were contoured by a clinical oncologist, in conjunction with an advising neuroradiologist and neurosurgeon where appropriate, incorporating possible microscopic spread where necessary. Adjacent OAR, such as brainstem, cochlea and optic chiasm, were contoured to ensure constrained doses to these structures.

**Figure 1:**
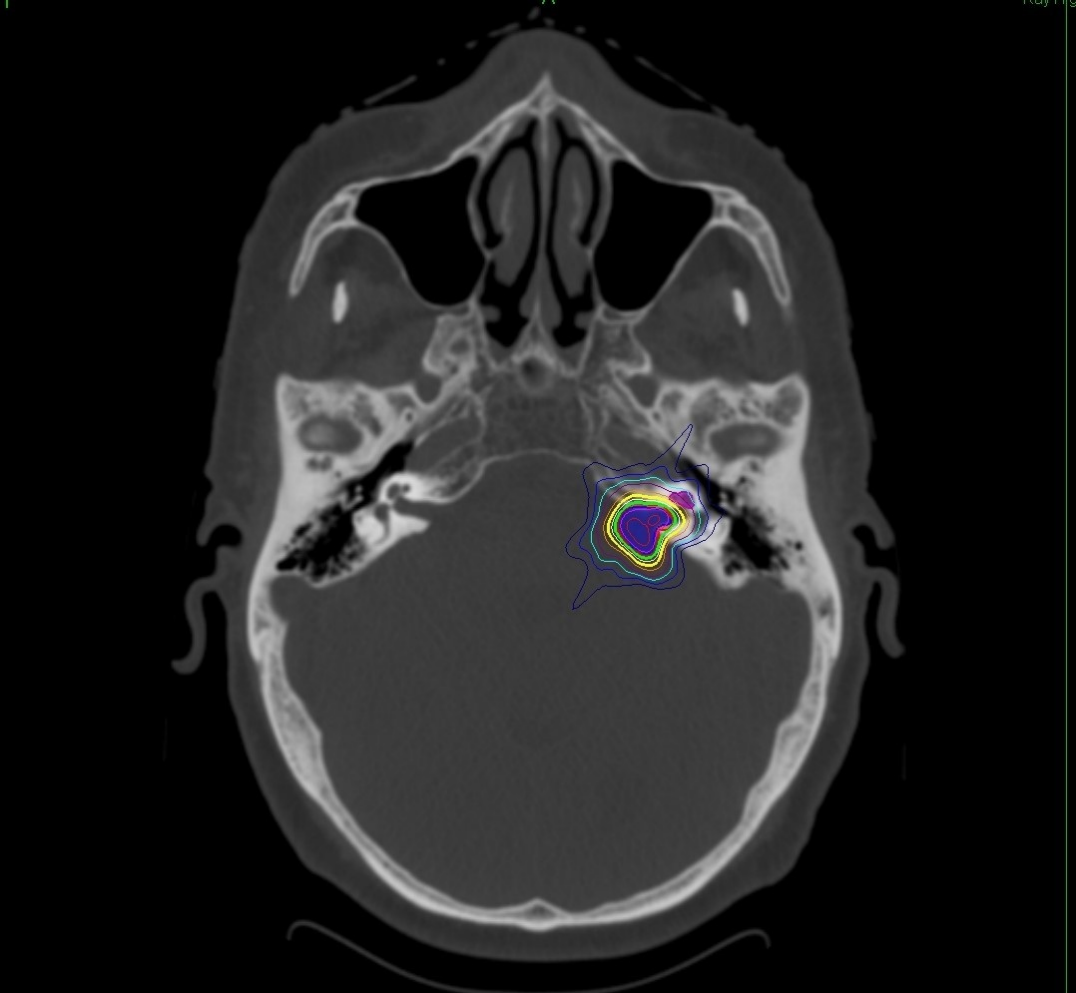
Treatment planning information from an example left-sided vestibular schwannoma patient. Axial computed tomography (CT) scan co-registered with magnetic resonance imaging (MRI) showing the treatment plan. The outlined tumor volume (blue shading) was expanded by 1.25 mm to create the planning target volume (PTV) (red shading). A high-degree of conformality was achieved between the PTV and the prescription isodose line (green contour line representing 2100 cGy). The surrounding isodose contours show sharp dose fall-off away from the tumor target with good sparing of the cochlea (magenta shading).

**Figure 2:**
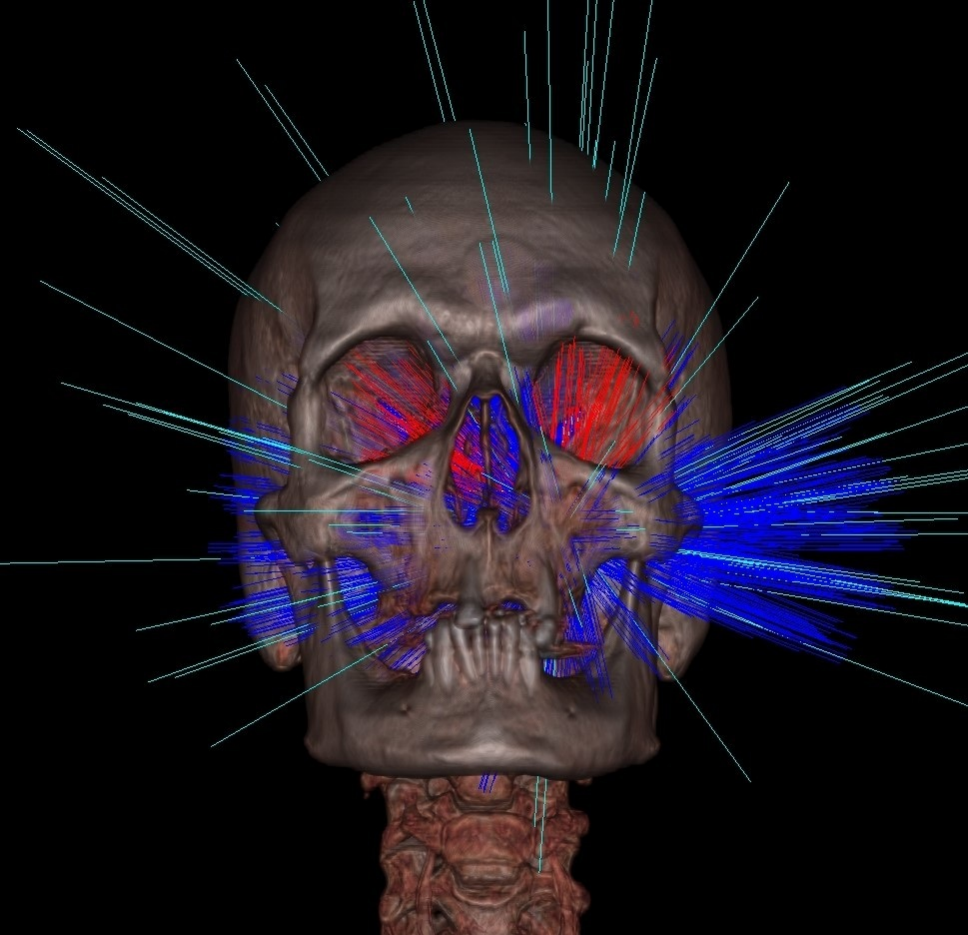
Treatment planning information from the same example left-sided vestibular schwannoma patient. Beam delivery plan illustrating the 95 beam trajectories selected for treatment.

Prescription dose, fractionation schedule, treatment margin and prescription isodose were decided on an individual patient basis, in accordance with prior published treatment recommendations, considering treatment intent, tumor type, tumor size, and the proximity of specific OAR and other critical structures [2-4,6,8,10-12,24-26]. In general, the following guiding principles were used to decide prescription dose: i) fractionation was the treating consultant’s choice, ii) fractionated treatment was usually preferable in order to minimize toxicity, unless toxicity was considered a lesser concern because of small tumor size and location sufficiently far from critical structures, iii) vestibular schwannomas were generally treated in three fractions, however, five fractions were used if there was a particular concern for toxicity, iv) treatment for vestibular schwannoma evolved with evidence that multi-fraction treatment with lower dose (18 Gy in three fractions) showed good tumor control and better hearing preservation than single fraction or higher dose treatment [8,25], iv) for meningiomas where the optic apparatus was considered at risk, treatment was delivered in five fractions [2,4,26]. For planning purposes, in most cases, a small safety margin (median 1.25 mm) was applied to the CTV to create the planning target volume (PTV) to account for potential sub-millimeter set-up error. In a minority of cases, the PTV was equivalent to the CTV and tumor targeting was optimized solely by isodose selection. Doses were prescribed to the PTV with the aim of achieving >95% target coverage. However, if an OAR constraint was compromised, coverage was maximized while keeping the OAR within tolerance. The optimal prescription isodose line was individually selected for each patient based on the assessment of multiple planning indices representative of dose distribution within and outside the target to maximize OAR sparing and target coverage (see Appendix for full details on individual patients' CTV-PTV margins, selected prescription isodose and PTV coverage). Treatment was delivered with a G4 CyberKnife® system using 6D Skull Tracking (Accuray Inc., Sunnyvale, USA). Fractionated treatments were administered on consecutive days where possible and prophylactic steroids were administered routinely from 2010 onwards [8,18]. Planning methodology images from an example patient are shown in Figure 1 and Figure 2.

Treatment planning indices were derived from the MultiPlan® system or subsequently calculated. New conformity index (nCI) was calculated using the formula nCI = TV x PIV/TV_PIV_^2^ , where TV = tumor volume (cc) and PIV = prescription isodose volume [27]. Biologically effective dose (BED) was calculated using the formula BED = D x [1+ d/α/β], where D = total dose and d = dose per fraction, assuming α/β = 2.4 Gy for schwannoma [24], 3.8 Gy for meningioma [24], 2.5 Gy for chordoma [3], 2.0 Gy for chondrosarcoma [28] and 3.0 Gy for paraganglioma [15].

Outcome data

Acute toxicity was reported by the treating consultant’s team at three to four weeks post-treatment, and graded in accordance with National Cancer Institute (NCI) Common Terminology Criteria for Adverse Events (CTCAE) version 4.0 [5]. Neurology assessment was restricted to the broad assessment of Nervous Systems Disorders section of CTCAE which covers cognitive disturbance and memory impairment. Late toxicity and outcome data were obtained from the referring consultants’ team at three, six, nine and 12 months after treatment and six monthly thereafter. Length of follow-up was defined as the time from the start of CyberKnife® treatment to the date of last patient clinical contact. Local progression-free survival (LPFS) was determined to either date of last contact or date of local treatment failure, and local progression was defined as radiological evidence of disease progression within, or adjacent to, the treatment site. Overall survival (OS) was determined by the date of last contact or date of death. All outcome data was analysed as an overall group of intracranial tumors and by pathological tumor sub-type. The rationale of the latter analysis was to take into account the tumor type characteristics such as typical size, radiosensitivity, α/β ratio and proximity to the specific OAR, and enable outcome comparisons of toxicity, local control, and survival to specific pathological tumor types in the scientific literature.

Statistical analysis

Chi-squared tests were performed to assess acute and late patient toxicity incidence (none, grade 1–2, and grade 3–4) in relation to PTV size (≤6.4 cc vs >6.4 cc). Kaplan-Meier analysis was performed to assess LPFS and OS. Log-rank analysis was used to compare LPFS in relation to PTV size.

## Results

Patients and treatment details

In total, 66 patients with skull-base tumors were consecutively treated in the period studied. This included patients with intracranial metastatic lesions, multiple tumors, recurrent malignant disease, tumors of uncertain pathology and benign neoplasms. Treatment intent was either palliative or curative, and varied from sole primary treatment to fourth-line therapy. Tumor types varied widely and included vestibular schwannoma, meningioma, chordoma, leiomyosarcoma, colon cancer metastases, craniopharyngioma, pituitary adenoma, chondrosarcoma, sarcoma, facial nerve schwannoma, facial neuroma, paraganglioma and uncertain pathological type. All patients received 15–30 Gy in one to five fractions: 43 patients (65%) were treated in three fractions, 15 patients (23%) were treated in five fractions and eight patients (12%) were treated in a single fraction. Fifty-eight patients were World Health Organization (WHO) performance status 0–1 and eight patients were WHO status 2–3. The median patient age was 54 years (range 23–83) and the median PTV was 6.4 cc (range 0.41–94.0).

A total of 13 patients were excluded from outcome analysis because they had multiple and/or secondary metastatic tumors. A further 18 patients were excluded because of missing data that precluded meaningful analysis: either because there was uncertainty regarding tumor histopathological type (only limited radiological diagnostic information was available for patients who had not undergone prior surgery, which in some cases was not definitive for tumor type) and/or because there was <2 weeks post-treatment follow-up information (unfortunately this was not uncommon for international patients and is acknowledged as a major study limitation).

The outcome analysis group therefore consisted of 35 patients with primary tumors of certain pathological diagnosis who were treated with curative intent, for whom reliable outcome information was available. Patient characteristics of the outcome analysis group had a similar median age (55 years), the same percentage of patients with WHO status 0–1 (88%), and the same median tumor PTV size (6.4 cc) compared with the overall treatment group. As per the overall treatment group, the outcome analysis patients also received 15–30 Gy in one to five fractions and the majority received fractionated treatment: 25 patients (71%) were treated in three fractions, eight patients (23%) were treated in five fractions and only two patients (6%) received treatment in a single fraction. Patient and tumor characteristics for the outcome analysis group are summarized in Table 1 and treatment information is given in Table 2. (Comprehensive individual patient data is given in Appendix 1.)

**Table 1:** Patient and tumor characteristics for outcome analysis patient group. WHO: World Health Organisation.

Characteristic	Category	Patients (n = 35)
Age	Median (range)	55 (24‒83) years
Gender	Female	16 (46%)
	Male	19 (54%)
WHO performance status	0‒1	31 (88.6%)
	2‒3	4 (11.4%)
Treatment indication	Primary treatment	17 (48.6%)
	Previous radiotherapy and surgery	3 (8.6%)
	Previous surgery	14 (40.0%)
	Previous radiotherapy	1 (2.8%)
Tumor type	Vestibular Schwannoma	16 (45.7%)
	Meningioma	12 (34.3%)
	Other:	7 (20.0%)
	Chordoma	2 (5.7%)
	Chondrosarcoma	2 (5.7%)
	Facial Nerve Schwannoma	1 (2.9%)
	Jugular Foramen Schwannoma	1 (2.9 %)
	Paraganglioma	1 (2.9%)

**Table 2:** Summary of treatment received for outcome study patient group. Median (range) values are given. PTV: Planning target volume; BED: Biological equivalent dose; nCI: new conformity index. *Margins and prescription isodoses were individually selected - in some cases no margin was applied and so lower isodose was selected for tumor targeting - see Appendix (Table 5) for individual patient information.

	PTV volume cc	Margin mm	PTV coverage %	Pres’n Isodose %	BED Gy_10_	nCI
All Tumors	6.4 (0.68–100.5)	1.25 (0–2.12) *	96.94 (79.6–99.5)	56.0 (46.0–71.0)*	82.3 (49.0–108.9)	1.3 (1.1–2.0)
Vestibular Schwannoma	3.8 (0.81–19.6)	1.25 (1.0–1.5)	97.7 (91.9–99.53)	55.0 (51.0–65.0)	82.3 (63.0–104.0)	1.3 (1.1–1.4)
Meningioma	5.5 (0.68–22.3)	1.25 (0–2.0)	91.9 (79.6–99.4)	60.0 (46.0–71.0)	67.1 (49.1–108.9)	1.4 (1.1–2.0)
Chondrosarcoma	8.2 (7.6–8.8)	1.32 (1.25–1.4)	96.2 (95.5–96.9)	57.0 (56.0–58.0)	90.0 (87.5–94.5)	1.5 (1.5–1.5)
Chordoma	66.5 (32.5–100.5)	1.0 (0–2.0)	97.20 (96.6–97.8)	63.5 (60.0–67.0)	90.0 (86.5–93.5)	1.3 (1.3–1.3)
Facial Nerve Schwannoma	11.0	1.0	98.1	65.0	82.5	1.3
Jugular Foramen Schwannoma	24.3	2.12	97.6	55.0	82.5	1.3
Paraganglioma	42.2	1.5	98.5	60.0	90.0	1.3

Acute toxicity

Acute and late toxicity data for the outcome analysis cohort is summarized in Table 3. Overall the treatment was well-tolerated with low incidence of acute toxicity that resolved within four weeks of treatment. Twenty-seven patients (77%) did not experience any acute side effects and eight patients (23%) experienced 15 incidences of toxicity: six patients (17%) reported grade 1–2 toxicities and two (6%) suffered grade 3–4 toxicities. The most common side effect was headache.

When analyzed by tumor type, only three of 16 vestibular schwannoma patients experienced toxicities: two (13%) experienced grade 1–2 toxicities and one patient (6%) suffered grade 4 headache, vomiting, and hydrocephalus. For meningioma patients, only two of 12 (17%) patients experienced grade 1–2 toxicities. For patients with ‘other’ tumor types, two of seven (29%) experienced grade 1–2 toxicities and one patient (14%) experienced grade 3 vomiting.

When all patients were considered and analyzed by tumor size, the incidence of acute toxicity was found to be unevenly distributed with tumor PTV size: tumors larger than the median (>6.4 cc) significantly associated with increased incidence and magnitude of acute toxicity (chi-squared p = 0.01). Notably, seven of the eight patients who experienced acute toxicities had tumors with PTV > 6.4 cc: two had vestibular schwannoma, which were 8.2 cc and 12.24 cc, respectively; two had meningiomas, which were 13.05 cc and 13.41 cc, respectively; and three had ‘other’ tumor types sized 8.7 cc, 11.4 cc, and 42.27 cc, respectively. Furthermore, both patients who experienced grade 3–4 toxicities had large tumors and other risk factors for increased toxicity. One patient with a large vestibular schwannoma (PTV = 12.24 cc) had previously received high dose cranial radiotherapy to a separate high-grade glioma. The patient was also the only patient in the cohort with WHO performance status 3. Despite being prescribed 21 Gy in three fractions over eight days to account for these adverse factors, the patient suffered grade 4 vomiting, headache and hydrocephalus. The second patient had a large paraganglioma (PTV = 42.2 cc) and was one of the first to be treated at our center. They received a single fraction of 15 Gy with no steroid prophylaxis and suffered grade 3 vomiting.

**Table 3:** Acute and late toxicity. CN: Cranial nerve.

Acute Toxicity						
Tumor Type	Patients	Symptom Incidence	Grade 1	Grade 2	Grade 3	Grade 4
Vestibular Schwannoma	N = 13	None	-	-	-	-
	N = 3	Paresthesia		1		
		Pain		1		
		Headache		1		1
		Vomiting				1
		Hydrocephalus				1
		Hearing Impairment	1			
Meningioma	N = 10	None	-	-	-	-
	N = 2	Headache		2		
		Tinnitus	1			
		Dizziness	1			
Other	N = 4	None	-	-	-	-
	N = 3	Headache		1		
		Fatigue		1		
		Balance		1		
		Vomiting			1	
Late Toxicity						
Tumor Type	Patients	Symptom Incidence	Grade 1	Grade 2	Grade 3	Grade 4
Vestibular Schwannoma	N = 8	None	-	-	-	-
	N = 8	Hearing loss			1	1
		Tinnitus	1			
		Pain	1			
		Fatigue	2			
		Nausea		1		
		Facial muscle weakness	1			1
		Dizziness/Vertigo	1			
		Oedema	1			
		Balance	1			
		Paresthesia		1		
		CN V/VII disorder	1	2		
		Tongue nerve disorder	1			
		CN VIII disorder			1	
		Hydrocephalus			1	
Meningioma	N = 4	None	-	-	-	-
	N = 8	Seizure	1			
		Memory impairment		1		
		Fatigue	2	1		
		Headache		3		
		Paresthesia		3		
		Alopecia	1			
		Facial muscle weakness	2	1		
		CN V/VII disorder	1	1		
		Hearing loss		1		
		Dizziness		1		
Other	N = 4	None	-	-	-	-
	N = 3	Seizure			1	
		Fatigue	1	1		
		CN V/VII disorder	2			
		Laryngeal palsy		1		

Late toxicity

Sixteen patients (46%) did not experience any late toxicity. The remaining 19 (54%) patients experienced a total of 44 incidences of late toxicity. The majority (86%) were grade 1–2 toxicities: only four patients (11%) experienced grade 3–4 toxicities. Overall, late toxicities were difficult to discriminate from tumor symptoms and in many cases represented stabilized or deteriorated pre-existing symptoms. The most common toxicities were fatigue and cranial nerve (CN) V/VII disorder. Only one patient reported memory impairment and there were no reports of cognitive dysfunction.

When analyzed by tumor type, eight of 16 (50%) vestibular schwannoma patients reported late toxicities: five (63%) reported grade 1–2 toxicities but three (38%) also experienced grade 3–4 toxicities consisting of CN disorders, hearing impairment and hydrocephalus. For meningioma patients, eight of 12 (67%) patients reported late toxicities, of which all incidences were grade 1–2. For patients with ‘other’ tumor types, three of seven (43%) patients reported late toxicities: two (67%) reported grade 1–2 toxicities only but one patient (33%) also experienced grade 3 seizure.

When analyzed by tumor size, the incidence of late toxicity did not correlate with PTV size. Instead, the more serious incidences of grade 3–4 late toxicity that occurred in four patients appeared to be related to anticipated increased toxicity risk or represented a deterioration of current symptoms. Three of the four patients who experienced serious late toxicities had known raised toxicity risk prior to treatment. The first patient had a large vestibular schwannoma (PTV = 19.6 cc) and was considered a high toxicity risk because of the size and location of the tumor to proximate critical structures. They were heavily counselled as a better candidate for surgery but opted for radiosurgery. Despite efforts to minimize toxicities by delivering 25 Gy in five fractions, with a protracted course over 14 days, the patient suffered serious late toxicities including deterioration of hydrocephaly. The second patient received treatment for a parasellar chondrosarcoma (PTV = 8.77 cc). The tumor was treated adjuvant to surgery to a total dose of 21 Gy in three fractions. This gave a relatively high BED of 94.5 Gy_10_ to maximize tumor control for this radio-resistant tumor, but concomitantly raised toxicity risk, and the patient suffered with late grade 3 seizure. The third patient had a small vestibular schwannoma (PTV = 1.56 cc) and received 21 Gy in three fractions, giving a BED of 82.3 Gy_10_. Small tumors of this type usually respond well to similar treatment [8,10,16,20]. However, in this case, the PTV encompassed the full length of the internal auditory canal and overlapped with a proportion of the cochlea structure. After treatment, late toxicities recorded for the patient represented further deterioration of pre-existing balance problems, acoustic nerve disorder and hearing impairment. Retrospective examination of the patient’s treatment plan showed that although the primary cochlea dose constraints were not exceeded (≤5 mm^3^ to receive ≥15 Gy), approximately 5% of the cochlea structure received 15 Gy (D5% = 15 Gy), approximately 1% of the cochlea volume received 18 Gy (D1% = 18 Gy) and the maximum point dose (MPD) was 19.4 Gy, indicating that several dose indices should be considered for small susceptible OAR. The fourth patient had a vestibular schwannoma (PTV = 3.8 cc) and experienced grade 3 hearing impairment post-treatment—however, this represented a relatively small worsening of hearing as the patient reported grade 2–3 hearing impairment as a pre-treatment symptom.

Effect of treatment on pre-existing symptoms

A total of 64 pre-existing and post-treatment symptom incidences were documented for the outcome analysis cohort. Data is summarized in Table 4. When analyzed by tumor type, for vestibular schwannoma patients, the most common pre-existing symptoms were hearing loss, tinnitus and balance problems: 71% of pre-existing symptoms were stable or improved post-treatment and 29% deteriorated. For meningioma patients, the most common pre-existing symptoms were eye and cranial nerve disorders: 88% of pre-existing symptoms were stable or improved post-treatment and 12% deteriorated. For other tumor types, the most common pre-existing symptoms were CN disorders: 66% of pre-existing symptoms remained stable post-treatment, but no symptoms were improved and 33% deteriorated.

**Table 4:** Pre- and post-treatment symptom incidence. CN: Cranial nerve.

Pre-treatment Symptom	Post-treatment Symptom Status		
	Deteriorated	Stable	Improved
Vestibular Schwannoma			
Hearing loss	3	7	2
Tinnitus	2	3	2
Balance	2	2	2
Dizziness/Vertigo	2	2	-
Nausea and vomiting	-	1	-
Headache/Localized pain	-	1	-
CN V/VII disorder	-	2	-
Hydrocephalus	1	-	-
Subtotal	10/34 (29%)	18/34 (53%)	6/34 (18%)
Meningioma			
Vision loss	1	3	-
Corneal reflex impaired		1	
Diplopia	-	-	1
Proptosis	-	1	
Eye pain	-	1	1
Headache/Localized pain	-	1	2
Hearing loss	-	2	1
Tinnitus	-	1	-
CN VIII/IV/VI disorder	-	1	-
CN V/VII disorder	2	3	
CN VIII disorder	-	2	-
Subtotal	3/24 (12%)	16/24 (67%)	5/24 (21%)
Other tumor types			
CN VIII/IV/VI disorder	-	1	-
CN V/VII disorder	-	2	-
CN VIII disorder	-	1	-
Neck pain	1	-	-
Balance	1	-	-
Subtotal	2/6 (33%)	4/6 (66%)	0/6 (0%)
TOTAL	14/64 (23%)	38/64 (59%)	11/64 (17%)

Local control and overall survival

Median follow-up time for the outcome analysis cohort was 13.4 months (range 0.87–51.4) and overall survival was 100% at one and two years. Local control data was available for 33 patients (no post-treatment radiological information available for two patients). Kaplan-Meier analysis showed overall LPFS was 95% at one year and 79% at two years. When analyzed by median tumor size, log-rank analysis showed LPFS was 100% at one and two years for tumors with PTV < 6.4 cc, but for tumors >6.4 cc local control reduced to 92% at one year and 72% at two years. However, this result may be influenced by tumor histopathological type, as all three of the patients who did show post-treatment local progression had ‘other’ tumor types (which were typically larger, more aggressive tumors), whereas no local progression was observed in patients with vestibular schwannoma or meningioma (which were typically smaller, benign tumors). For vestibular schwannoma and meningioma, the crude rate of local control was 100% at median follow-up of 11.4 months (range 0.87–26.9) and 22.1 months (range 6.0–51.4), respectively. Whereas for ‘other’ tumor types, the crude rate of local control was 43% at median follow-up of 17.7 months (range 7.3–26.4).

All three patients who progressed locally in the follow-up period had larger tumors and had previously undergone surgery. The first patient had a large clival chordoma (PTV = 100.46 cc) which showed progression within the treated area 18.8 months post-treatment. The second patient had a jugular foramen schwannoma (PTV = 24.25 cc) and showed an enlarging cyst adjacent to the tumor 10.1 months post-treatment. Both patients received 21 Gy in three fractions and experienced no acute or late toxicities. The third patient had a parasellar chondrosarcoma (PTV = 8.8 cc) which showed a small area of growth adjacent to the treatment site between the basilar artery and the brainstem 17.5 months post-treatment. This patient received 21 Gy in three fractions and experienced grade 3 seizure as late toxicity.

## Discussion

This study represents the first patients in the UK to receive CyberKnife® treatment for skull-base tumors and consists of a broad patient cohort with heterogeneous tumor types that included many challenging patient cases. The results obtained illustrate the importance of taking tumor type and size into account throughout the treatment process and in the assessment of treatment outcome, supporting previous reports that tumor type and size influence outcome for base-of-skull radiosurgery [1-7,10-14,18,23]. The main limitation of the study was the restricted outcome analysis group, because of the limited availability of diagnostic tumor type information and follow-up data. The tumor type information was limited because only radiological information was available for patients who had not undergone prior surgery, and this was indefinite for tumor histopathological type in some cases. The follow-up data was limited largely because of the nature of the treatment center. As one of the first clinics to offer stereotactic radiosurgery, international patients would often travel to the clinic for specialist short-term treatment, and then return to their home countries. As a result, although attempts were made to acquire first-hand clinical assessments of outcome, or to retrieve subsequent follow-up data from referring clinicians, this was not achieved in many cases. In hindsight, it is acknowledged that a prospective arrangement for follow-up, such as maintaining direct patient contact through post-treatment telephone consultation [29], would have improved the quantity and quality of post-treatment data collection.

Generally, radiosurgery was well tolerated. For meningioma patients, acute toxicity was lower than a previous retrospective review of Cyberknife® treatment in meningiomas of very similar size [26]. For vestibular schwannoma patients, acute toxicity was also encouragingly low, in accordance with previous reports of radiosurgery being ‘well tolerated’ [8], although there is a lack of acute toxicity information for meaningful comparison [6,9,12,29]. Increased incidence of acute toxicity was significantly associated with tumor size >6.4 cc, a finding that fits well with previous studies that have associated tumors >8.0 cc with increased toxicity [11,12,27]. Notably, both patients who experienced grade 3–4 acute toxicity were anticipated to be at higher risk of acute toxicity because they had large tumors. The first patient (who had large vestibular schwannoma PTV = 12.24 cc) had further toxicity risk, as they had poor WHO performance status and had previously received high-dose cranial radiotherapy. Although fractionated treatment was delivered in three fractions over a protracted course, the patient suffered grade 4 acute toxicities. It is likely that treatment in five fractions, and/or a reduction in overall dose, and/or deferring the treatment may have reduced the toxicities while still maintaining good local control [8,25]. The second patient (who had a large paraganglioma PTV = 42.2 cc) received single fraction treatment with no steroid prophylaxis and suffered grade 3 acute vomiting. In accordance with our developed guidelines, prophylactic medications are now routinely used, and a similar patient would now receive fractionated treatment to minimize toxicity. It is interesting to note that the other patient who received single fraction treatment did not experience any toxicities—this is likely because the patient had a small olfactory groove meningioma (PTV = 2.6 cc) that was located sufficiently far from critical structures such that toxicity risk was considered to be low, supporting data that single fraction treatment is better for smaller tumors at lower risk locations [4, 8]. Our experience with the two patients who experienced serious acute toxicities highlighted the need for careful patient counselling where risk is high, as well as the importance of practices to minimize acute side effects, such as evolving recommendations for prescription doses and fractionation for specific tumor types and locations—all of which have now been incorporated into our current treatment guidelines.

The assessment of late toxicity was hampered by difficulty discriminating between pre-treatment symptoms, which were derived from patient notes, and post-treatment side-effects, which were derived from clinical assessment by the referring consultant’s treatment team. Many recorded late toxicities represented a deterioration of pre-treatment symptoms rather than a newly arising treatment toxicity, and in some cases may have been due to tumor progression rather than treatment-related side-effects. Thus, both the incidence and magnitude of treatment-related late toxicities are likely to have been exaggerated. Direct patient contact by the treating team, and a more objective evaluation of pre- and post-treatment symptoms and side-effects, e.g., hearing function using audiograms and speech discrimination [18] and tabulated grading of facial and visual function, would have been beneficial. Late toxicity was experienced by a relatively high proportion of patients (54%), but was low grade for most patients. Only four (11%) patients suffered late grade 3–4 toxicities, where in each case lessons may be learnt for toxicity incidence. A higher risk of side-effects was anticipated in three patients because of individualized tumor factors, such as large tumor proximate to OAR, radioresistant tumor type necessitating high radiation dose for control, or target tissue overlapping susceptible OAR. One patient with large vestibular schwannoma (PTV = 19.6 cc) was not considered a good candidate for stereotactic radiotherapy and indeed, despite considerable efforts to minimize toxicity (25 Gy in five fractions delivered over 14 days), the patient suffered serious morbidities, exemplifying the need for careful patient selection. The second patient had a parasellar chondrosarcoma (PTV = 8.77 cc) which was treated with 21 Gy in three fractions to maximize tumor control adjuvant to surgery. However, because of the relatively high BED (94.5 Gy_10_), in hindsight increased fractionation may have reduced morbidity. The third patient had a small vestibular schwannoma (PTV = 1.56 cc) and received 21 Gy in three fractions, giving a BED of 82.3 Gy_10_ and was expected to respond well [8,10,16,20]. However, in this case the PTV overlapped a proportion of the cochlea structure and the patient experienced deterioration of pre-existing balance problems, acoustic nerve disorder, and hearing impairment post-treatment. Retrospective consideration of the patient case indicated that several dose indices, such as D1%, D5% and MPD, are more informative for small susceptible OAR like the cochlea, to enable a more comprehensive picture of risk. The fourth patient had a vestibular schwannoma and received 21 Gy in three fractions. Their post-treatment hearing impairment was recorded as a serious side-effect, although it represented a marginal worsening of significant pre-treatment hearing impairment. Nevertheless, the serious toxicity experienced by two vestibular schwannoma patients provided evidence that a reduction in overall dose to 18 Gy with fractionated delivery may help hearing and neurological preservation [8,25]—a practice we have now adopted for vestibular schwannoma patients. Our institutional practices have also changed regarding cochlea sparing, where our planning practices have become more meticulous and now include assessment of several cochlea dose indices to optimize outcome.

Encouragingly, 76% of all pre-treatment symptom incidences were stable or improved post-treatment. This compares well with reported rates of neurological function preservation rates of 50–82% for schwannoma [14,17,18, 30], 74–96% for meningioma [1,2,5,11] and 78% for chondrosarcoma and chordoma [10] using radiosurgery approaches. For vestibular schwannoma patients, we found nine of 12 (75%) patients had preserved or improved hearing loss post-treatment, which compares favorably with other literature [9,12,17,18,30]. For meningioma patients, 21/24 (88%) of pre-existing symptoms remained stable or improved, again comparing favorably with other data [5,11,23,26]. For patients with other tumor types, neurological function was maintained in four of six patients (67%): however, neck pain deteriorated in one patient with large clival chordoma (PTV = 100.46 cc) and balance deteriorated in one patient with left facial nerve schwannoma (PTV = 11.04 cc).

Local control also compared well with other studies of radiosurgery in cranial tumors [3-10,12,13,16,18,21,22]. Although only limited to two-year assessment, local control for benign vestibular schwannomas and meningiomas was 100% at two years, which compares favorably with other two-year local control rates of 91–100% [6,8,14,29] and 69–100% [1,2,4,5,11,16,26], respectively. Notably all incidences of local progression occurred in ‘other’ tumor types, all three of which had undergone prior surgery, had larger PTV associated with reduced local control [6,11-13], or were a type of tumor known to be relatively radio-resistant and prone to recurrence [3,7,10]. Nevertheless, examination of the individual patient cases indicates room for improvement. The first patient who showed local progression had a large chordoma (PTV = 100.46 cc), which was a recurrent tumor that was previously treated with radiation therapy and had been surgically addressed on three separate occasions. Because of toxicity risk, the patient was prescribed 22 Gy in three fractions, resulting in relatively low BED of 86.0 Gy_10_. However, as the patient experienced no acute or late toxicities, they may have benefited from the increased dose in five fractions to increase BED and optimize local control [3]. The second patient who showed local progression had a large jugular foramen schwannoma (PTV = 24.24 cc) and had previously undergone sub-total surgical excision. This is consistent with a previous study reporting both previous surgery and tumor size >10 cc to be significant independent adverse factors for schwannoma tumor control [12]. Again, as the patient experienced neither acute nor late toxicities after 21 Gy in three fractions, it may have been possible to increase their overall treatment dose, with increased fractionation to limit toxicity. The third patient was the parasellar chondrosarcoma patient in whom tumor progression due to an adjacent small area of growth was observed 17.5 months post-treatment, who also suffered serious late toxicity. This indicates that overall dose may need to be increased to achieve better control in this radio-resistant tumor type, delivered in a greater number of fractions to limit toxicity. The crude rate of local progression in our chordoma and chondrosarcoma were therefore two of four (50%), which is lower than previous reports of 59–89% [3,7,10]. This indicates the need for further optimization of planning dosimetry methodologies and fractionation scheduling to improve the radiosurgical treatment in these notoriously difficult radio-resistant tumors: nevertheless, the lack of any toxicities in the two chordoma patients and one of two chondrosarcoma patients are encouraging.

## Conclusions

Our experience has confirmed that CyberKnife® radiosurgery for skull-base tumors is well-tolerated and effective. The results obtained support other studies that have related treatment success to tumor histological type and size, and demonstrate the need to learn from prior experiences to shape ongoing best practice.

For smaller radiosensitive tumors, such as vestibular schwannoma and meningioma, radiosurgery resulted in minimal acute toxicity and excellent local control. The main areas requiring improvement were symptom management and late morbidity: as such, neurological preservation and symptomatic amelioration should become the focus of treatment improvements for these tumor types. For vestibular schwannoma patients, our results indicated greater treatment-related toxicity for patients with larger tumors, those who received less optimal dose/fractionation schedule, or those who had other risk factors, such as previous cranial radiotherapy or PTV that abutted the cochlea. Optimal dose prescription and fractionation, and vigilance of multiple dose indices for susceptible OAR at planning, may aid reduction of late toxicity and optimize functional preservation. For tumors of other pathological types, which tend to be larger and/or more radioresistant, our results indicate efforts to increase local control with concomitant minimization of toxicity, through optimization of dose and fractionation scheduling, which are the first steps to improve treatment outcome.

## Appendices

**Table 5:** Supplementary patient data. F: Female; M: Male; WHO PS: World Health Organisation performance status; RT: Radiotherapy; Tx: Treatment; PTV: Planning target volume; CPA: Cerebellopontine angle; CTV: Clinical target volume; P'n: Prescription; nCI: new conformity index; HI: Homogeneity index; BED: Biological equivalent dose; I: Improved; S: Stable; D: Deteriorated; CN: Cranial nerve; RHS: Right hand side; n&v: Nausea and vomiting; ND: Nerve disorder; UK: Unknown.

Patient	Treatment Site	Subsite	Type	Gender	WHO PS	Tumor Details	Prev. Surg.	Prev. RT	Age@Tx	PTV cc	PTV < 6.39 cc	Margin (mm)	CTV	P'nDosecGy	P'nIsodose%	Fractions	mindosetoPTVcGy	maxdosetoPTV cGy	meandosetoPTVcGy	nCI	HI	% cover	BED	Acute Toxicity Summary	Pre-existing Symptom and Post-treatment Status I/S/D	Local Prog	FU_months (txtodatelastseen)	Late Toxicity Summary	NOTES
1	Intracranial	Skull base	VESTIBULAR SCHWANNOMA	F	1	Acoustic neuroma	0	0	66.99	19.62	1	1	15.885	2500	56	5	1796.47	4464.29	3209.09	1.21	1.79	92.36	77.03	none	hydrocephalus (D)	0	12.30	facial muscle weakness (G4), trigeminal ND (G1), tongue ND (G1), hydrocephalus (G3)	
2	Intracranial	Skull base	VESTIBULAR SCHWANNOMA	M	1	Acoustic neuroma	0	0	46.84	2.21	0	1.25	1.48	2100	56	3	1946.16	3750	2737.57	1.17	1.79	98.04	82.50	none	tinnitus (I) and hearing loss (I)	0	10.00	none	
3	Intracranial	Skull base	VESTIBULAR SCHWANNOMA	M	1	Acoustic neuroma	0	0	48.47	0.81	0	1.25	0.43	2100	61	3	1132.97	3442.62	2655.48	1.31	1.64	95.39	82.50	hearing impaired (G1)	dizziness (D), hearing loss (D), and balance problems (D)	0	4.10	none	
4	Intracranial	Skull base	VESTIBULAR SCHWANNOMA	M	1	Acoustic neuroma	0	1	50.85	1.08	0	1.25	0.59	2100	59	3	1248.18	3559.32	2715.21	1.27	1.69	97.98	82.50	none	reduced hearing (I)	0	13.43	balance (G1)	previous cranial irradiation (childhood leukaemia)
5	Intracranial	Skull base	VESTIBULAR SCHWANNOMA	F	2	Left acoustic neuroma	0	0	72.33	5.6	0	1.5	3.65	1800	50	3	1267.55	3600	2544.84	1.23	2	95.88	63.00	none		0	26.90	fatigue (G1),nausea (G2)	
6	Intracranial	Skull base	VESTIBULAR SCHWANNOMA	F	2	Acoustic neuroma	0	0	83.04	1.98	0	1.25	1.21	2100	65	3	2006.83	3230.77	2651.88	1.13	1.54	98.03	82.50	none	complete hearing loss pre-Tx (S), balance problems (S), hemi-facial spasm (CN disord. V/VII) (S)	0	10.90	oedema (G1)	complete hearing loss pre-treatment
7	Intracranial	Skull base	VESTIBULAR SCHWANNOMA	M	3	Acoustic neuroma	0	0	59.19	12.24	1	1.25	9.84	2100	53	3	1083.81	3962.26	2880.76	1.33	1.89	91.85	82.50	headache (G4), vomiting (G4), hydrocephalus (G4)	balance (I)	0	19.87	none	previous cranial irradiation (to separate high grade glioma)
8	Intracranial	Skull base	VESTIBULAR SCHWANNOMA	M	1	Right vestibular schwannoma	0	0	60.56	10.0	1	1	7.89	2100	53	3	1161.83	3962.26	2867.76	1.16	1.89	97.58	82.50	none	deaf in RHS (S)	0	12.20	facial muscle weakness (G1)	
9	Intracranial	Skull base	VESTIBULAR SCHWANNOMA	M	1	Acoustic neuroma	0	0	52.57	9.66	1	1.25	7.66	2400	55	3	1635.45	4363.64	3225.52	1.3	1.82	94.97	104.00	none	dizziness (S), hearing loss (S), vertigo (S), tinnitus (S), n&v (S), headache (S), balance (S)	0	14.03	none	
10	Intracranial	Skull base	VESTIBULAR SCHWANNOMA	M	1	Left vestibular schwannoma	1	0	64.67	8.27	1	1	6.20	2100	55	3	1156.5	3818.18	2751.99	1.3	1.82	95.75	82.50	parathesia (G2) pain (G2)	balance (I), hearing loss (S), tinnitus (D)	0	9.83	dizziness (G1)	
11	Intracranial	Skull base	VESTIBULAR SCHWANNOMA	M	1	Left acoustic neuroma	0	0	54.52	3.77	0	1	2.65	2100	55	3	1287.11	3818.18	2797.02	1.34	1.82	95.16	82.50	none	hearing loss (D), tinnitus (D)	0	13.47	hearing (G3), tinnitus (G1)	
12	Intracranial	Skull base	VESTIBULAR SCHWANNOMA	M	1	Left acoustic neuroma	0	0	66.79	~0.95	0	1.01	0.53	2100	54	3	2072.03	3888.89	2798.74	1.14	1.85	99.53	82.50	none	tinnitus (S) and hearing loss (S)	UK	3.23	none	
13	Intracranial	Skull base	VESTIBULAR SCHWANNOMA	M	1	Acoustic neuroma	0	0	65.83	5.87	0	1.25	0.28	2100	55	3	1720.07	3818.18	2762.99	1.21	1.82	98.54	82.50	none	hearing loss (S)	0	0.87	none	
14	Intracranial	Skull base	VESTIBULAR SCHWANNOMA	M	1	Acoustic neuroma	1	0	44.04	0.98	0	1.25	0.50	2100	62	3	1931.71	3387.1	2716.83	1.26	1.61	98.45	82.50	none	none (S)	0	1.77	none	
15	Intracranial	Skull base	VESTIBULAR SCHWANNOMA	F	1	Right IAM	0	0	58.49	0.91	0	1.25	0.51	2100	56	3	1705.71	3750	2842.08	1.15	1.79	98.77	82.50	none	tinnitus (S), RHS hearing loss (S), vertigo (D)	0	11.90	none	
16	Intracranial	Skull base	VESTIBULAR SCHWANNOMA	F	1	Acoustic neuroma	0	0	61.34	1.56	0	1.25	0.97	2100	51	3	1111.22	4117.65	2964.17	1.44	1.96	97.72	82.50	none	hearing loss (D), vertigo (S) tinnitus (I), balance (D), facial dyskinesis (CN disord. V/VII) (S)	0	10.00	fatigue (G1), pain (G1), paresthesia (G2), acoustic ND (G3), facial ND (G2), trigeminal ND (G2), hearing impairment (G4)	
17	Intracranial	Skull base	MENINGIOMA	M	1	Clival meningioma	1	0	55.75	1.04	0	1.25	0.58	2400	66	3	1313.03	3636.36	2731.68	1.33	1.52	86.76	65.20	none	left eye pain (I)	0	15.93	none	
18	Intracranial	Skull base	MENINGIOMA	F	1	Right sphenoid wing meningioma	1	0	41.10	13.05	1	0	13.05	2500	61	5	2372.33	4098.36	3004.7	1.51	1.64	89.48	57.89	headache (G2),	visual loss (d) headache (i)	0	51.40	fatigue (G1), headache (G2)	
19	Intracranial	Skull base	MENINGIOMA	M	1	Meningioma	1	0	55.90	3.19	0	2	1.46	3000	60	3	2956.21	5000	3836.92	1.14	1.67	99.43	108.95	none		UK	47.53	fatigue (G2)	
20	Intracranial	Skull base	MENINGIOMA	F	1	Foramen magnum meningioma	1	0	35.22	5.92	0	1	4.67	2400	71	3	2040.77	3380.28	2780.88	1.29	1.41	97.66	65.20	none	corneal reflex (s); nystagmus (CNIII, IV, VI) (s); CN V (S) VII (S) and VIII (s); headache (i)	0	42.33	headache (G2), parasthesia (G2), weakness (G1), alopecia (G1)	
21	Intracranial	Skull base	MENINGIOMA	F	1	Skull base meningioma	1	0	69.98	3.40	0	0	3.40	2250	52	5	1301.35	4326.92	3190.19	1.47	1.92	92.09	49.15	none	visual loss due to surgery (S)	0	16.33	seizure (G1), memory impairment (G2)	
22	Intracranial	Skull base	MENINGIOMA	M	1	Right CPA meningioma	1	1	48.85	22.29	1	1.65	16.78	1800	46	5	159.48	3913.04	2490.25	2.04	2.17	79.58	35.05	none	deafness (S) CN V symptoms (D)	0	23.47	trigeminal ND (G2)	complete hearing loss on RHS pre-treatment
23	Intracranial	Skull base	MENINGIOMA	F	1	Left CPA meningioma	0	0	46.45	13.41	1	1.5	9.58	2100	58	3	1264.74	3620.69	2585.02	1.37	1.72	83.86	59.68	tinnitus (G1), headache (G2), dizziness (G1)	trigeminal (CN V) nerve disorder (D); CN VII symptoms (S); CN VIII symptoms (S)	0	35.77	fatigue (G1), paresthesia (G2), facial muscle weakness (G2), hearing impaired (G2), dizziness (G2)	
24	Intracranial	Skull base	MENINGIOMA	F	1	Left petrous apex meningioma	1	0	40.39	5.15	0	1.25	3.38	2250	64	5	2008.74	3515.63	2634.59	1.5	1.56	86.9	49.15	none	hearing loss (I)	0	13.43	none	
25	Intracranial	Skull base	MENINGIOMA	F	0	Olfactory groove meningioma	1	0	40.98	2.55	0	2	0.9204	1700	60	1	1633.98	2833.33	2197.82	1.2	1.67	98.4	93.05	none	hearing loss (S) and tinnitus (S)	0	23.00	none	
26	Intracranial	Skull base	MENINGIOMA	F	1	Menigioma	0	0	50.30	7.61	1	1	4.99	2500	61	5	1051.76	4098.36	3092.28	1.36	1.64	95.44	57.89	none	visual loss (S) proptosis (S) double vision (I) pain in eye (S) headache (S)	0	21.20	paresthesia (G2), facial muscle weakness(G1), facial ND (G1)	
27	Intracranial	Skull base	MENINGIOMA	F	1	Petrous apex menigioma	0	0	67.98	6.85	1	1.25	5.20	2400	54	3	1337.47	4444.44	3331.54	1.35	1.85	91.76	65.20	none		0	6.00	headache (G2)	
28	Intracranial	Skull base	MENINGIOMA	F	1	Meningioma	1	0	71.16	0.68	0	1.25	0.43	2400	52	3	2288.41	4615.38	3228.79	1.28	1.92	98.71	65.20	none	visual loss (S)	0	9.40	none	
29	Intracranial	Skull base	OTHER CHORDOMA	M	1	Clival chordoma	1	1	59.89	100.46	1	2	75.06	2200	60	3	1534.33	3666.67	2740.72	1.32	1.67	96.61	86.53	none	neck pain (D)	1	18.80	none	
30	Intracranial	Skull base	OTHER CHORDOMA	M	2	Left temporal chordoma	1	1	65.82	32.5	1	0	32.50	2850	67	5	2274.14	4253.73	3400.03	1.34	1.49	97.78	93.48	none		0	24.93	none	
31	Intracranial	Skull base	OTHER SCHWANNOMA	M	1	Left facial nerve Schwannoma	1	0	58.11	11.04	1	1	8.25	2100	65	3	1590.53	3230.77	2496.75	1.26	1.54	98.09	82.50	balance (G2)	CN V (S) Balance (D)	0	7.27	facial muscle weakness (G1), facial ND (G1), trigeminal ND (G1)	
32	Intracranial	Skull base	OTHER SCHWANNOMA	M	1	Jugular foramen schwannoma	1	0	40.48	24.25	1	2.12	14.93	2100	55	3	831.63	3818.18	2791.34	1.26	1.82	97.6	82.25	none		1	10.10	none	
33	Intracranial	Skull base	OTHER PARAGANGLIOMA	F	1	Skull base paraganglioma	0	0	43.63	42.19	1	1.5	26.1	1500	60	1	795.57	2500	1885.23	1.27	1.67	98.5	90.00	vomiting (G3)		0	26.03	fatigue (G1), laryngeal palsy (G2)	
34	Intracranial	Skull base	OTHER CHONDROSARCOMA	M	1	Clivus sarcoma	1	0	60.76	7.63	1	1.25	5.39	2500	58	5	1853.52	4310.34	3314.34	1.46	1.72	95.45	87.50	none	CN V/VII (S) CN VIII (S), visual cranial nerves (VIII/IV/VI) (S)	0	12.27	none	
35	Intracranial	Skull base	OTHER CHONDROSARCOMA	F	1	Parasellar region chondrosarcoma	1	0	24.37	8.77	1	1.38	6.06	2100	56	3	1370.33	3750	2910.59	1.57	1.79	96.94	94.50	headache (G2), fatigue (G2),		1	17.53	fatigue (G2), seizure (G3)	
